# Supplementary figures and images for: Point-estimating observer models for latent cause detection
Source: PLoS Comput Biol. 2021 Oct 29;17(10):e1009159. doi: 10.1371/journal.pcbi.1009159 (PMC8580258; doi:10.1371/journal.pcbi.1009159)

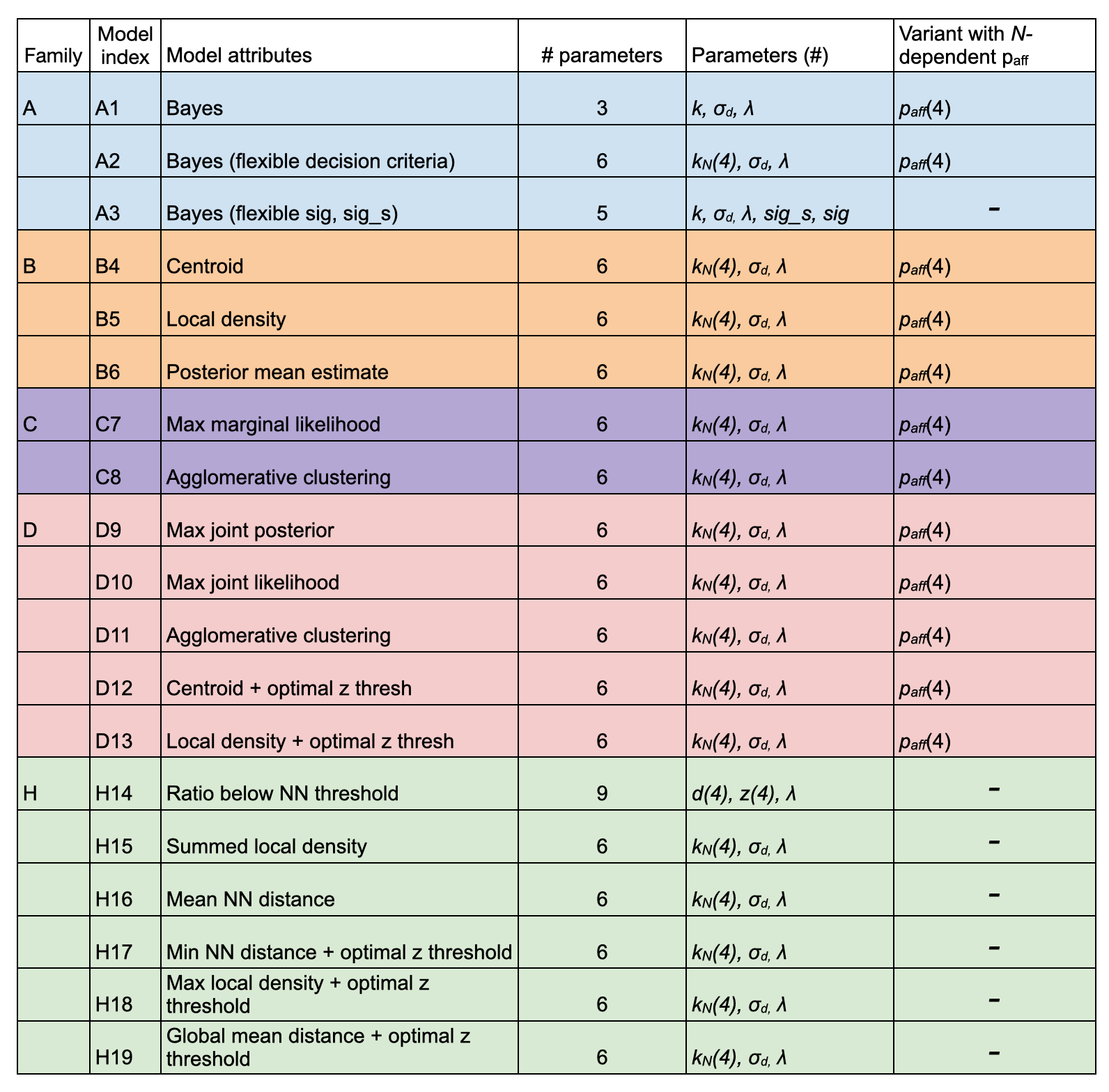

Supplement: S1 Fig — Model parameters. N-dependent parameters are denoted by ‘(4)’, reflecting a unique parameter fit for each N = 6, 9, 12, 15 condition. (TIFF) [file pcbi.1009159.s001.tiff]

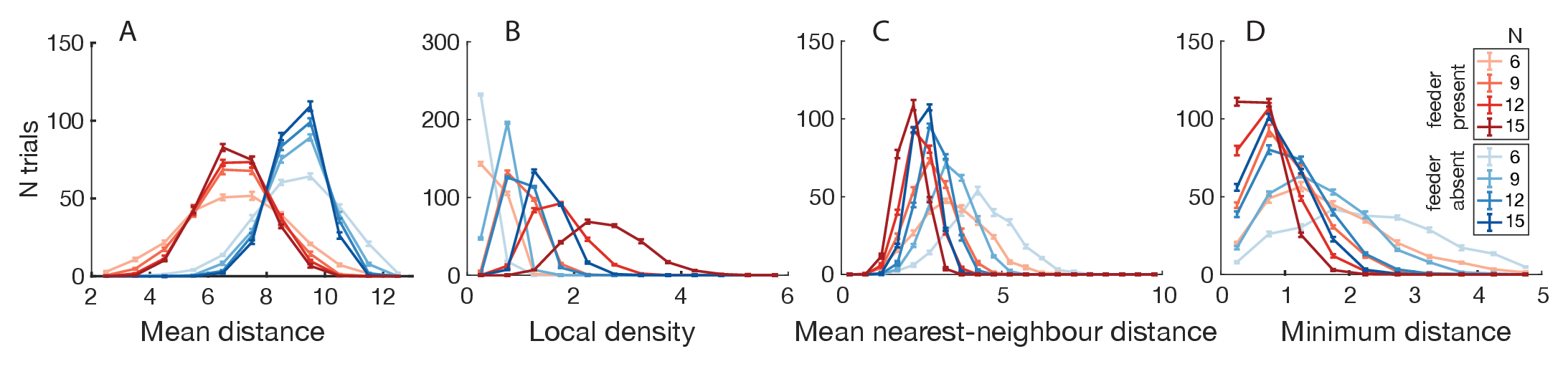

Supplement: S2 Fig — Stimulus histogram distributions along each distance-based heuristic. “Feeder absent” trials in blue, “feeder present” trials in red, with colour saturation indicating the number of pigeons on a given trial (N = 6, 9, 12, 15). For each trial, the following quantities were computed: the mean pairwise distance of all pigeons (A), the maximum local density, where local density is computed by convolving a gaussian of σ = 1.4cm over the circular arena (B), the nearest neighbour distance, calculated as the mean distance between all pigeons and their nearest neighbour (C), and the distance between the two nearest pigeons (D). (TIFF) [file pcbi.1009159.s002.tiff]

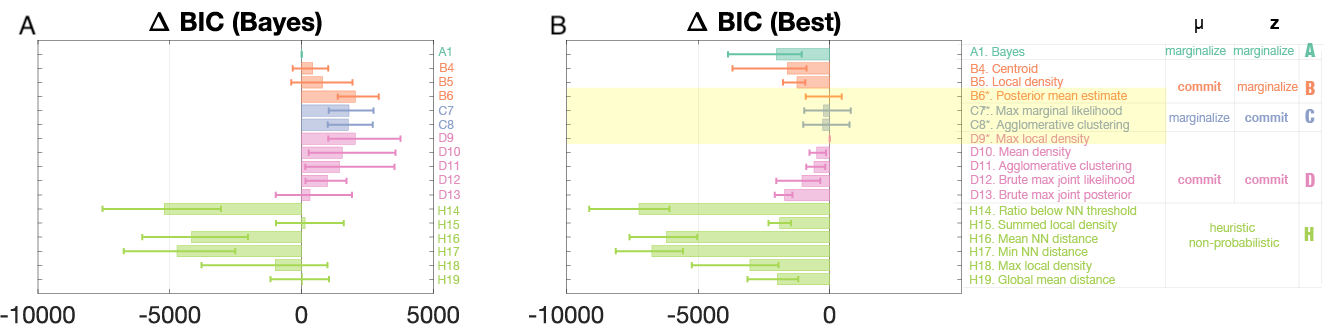

Supplement: S3 Fig — Model comparison (Bayesian Information Criterion (BIC)). Compared to AIC, BIC model comparison penalizes model complexity more heavily. MML denotes “Maximum Marginal Likelihood,” MMP denotes “Maximum Marginal Posterior,” MJP denotes “Maximum Joint Posterior.” NN denotes “Nearest Neighbour.” The difference in BIC scores between models (Δ BIC) plotted provides an estimate of the quality of each model relative to the fully Bayesian model (A1) in panel A, or the best-fitting model (D9) in panel B. The four best models (highlighted) are indistinguishable via AIC and BIC. (TIF) [file pcbi.1009159.s003.tif]

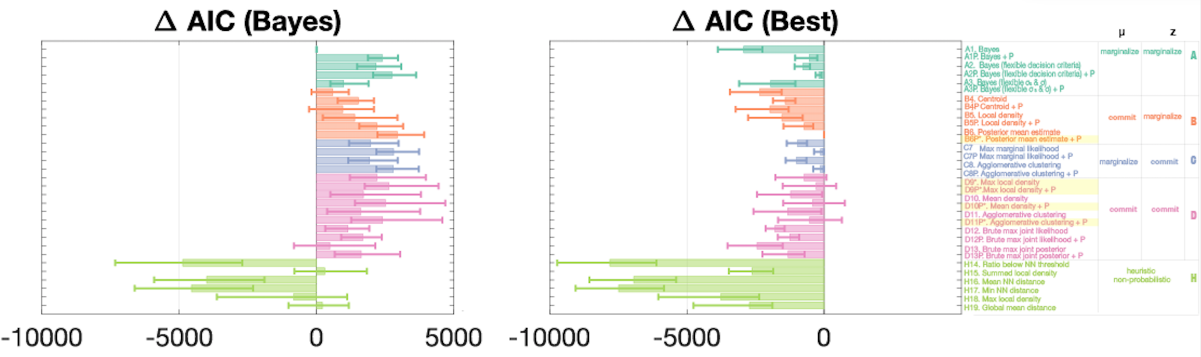

Supplement: S4 Fig — Model comparison (Bayesian Information Criterion (BIC)) with probability of affiliation fitted as a separate free parameter for each of the four N conditions (N = 6, 9, 12, 15) are denoted with the addition of “P” to the model name. Compared to AIC, BIC model comparison penalizes model complexity more heavily. MML denotes “Maximum Marginal Likelihood,” MMP denotes “Maximum Marginal Posterior,” MJP denotes “Maximum Joint Posterior.” NN denotes “Nearest Neighbour.” The difference in BIC scores between models (Δ BIC) plotted provides an estimate of the quality of each model relative to the fully Bayesian model (A1) in panel A, or the best-fitting model (B6P) in panel B. Adding a flexible N-dependent criterion can help rescue the Bayesian model (see model A2 compared to A1), and adding 4 flexible N-dependent probability of affiliation parameters increases goodness of fit for all models. (TIF) [file pcbi.1009159.s004.tif]

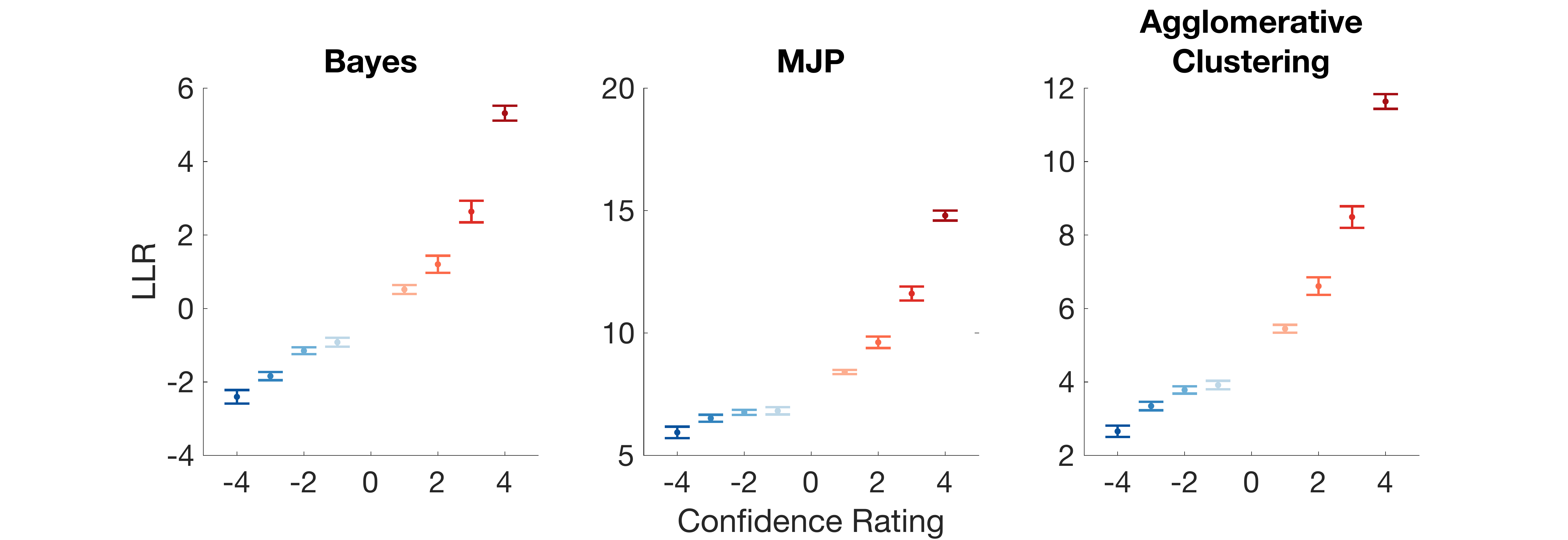

Supplement: S5 Fig — Average d (log likelihood ratios) for trials binned by response x confidence pairs across all subjects. Negative confidence rating denotes “feeder absent” response and positive confidence denotes a “feeder present” response. (TIFF) [file pcbi.1009159.s005.tiff]

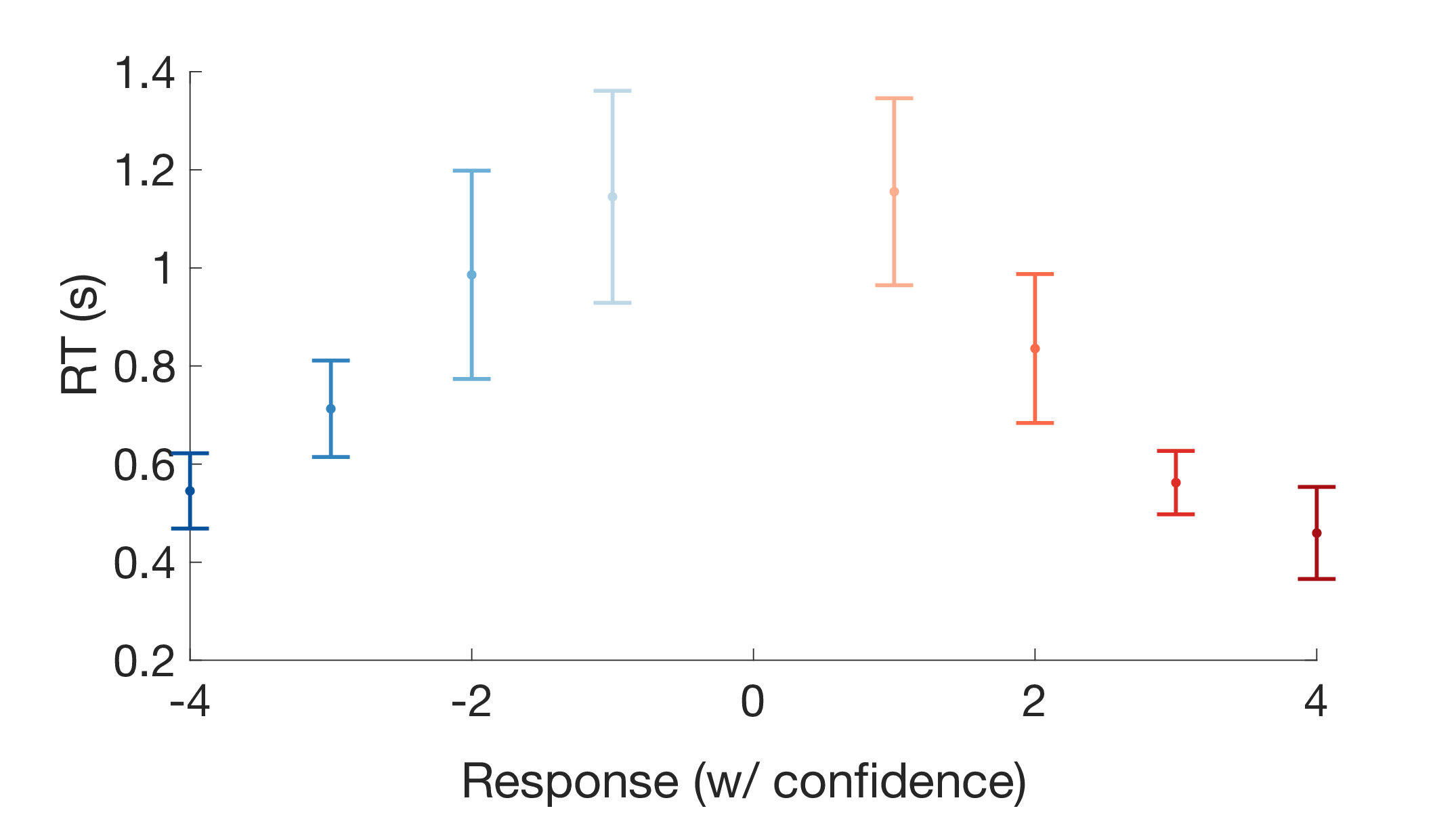

Supplement: S6 Fig — Subject reaction times for each response x confidence pair, where negative confidence denotes a “feeder absent” response and positive confidence denotes a “feeder present” response. (TIFF) [file pcbi.1009159.s006.tiff]

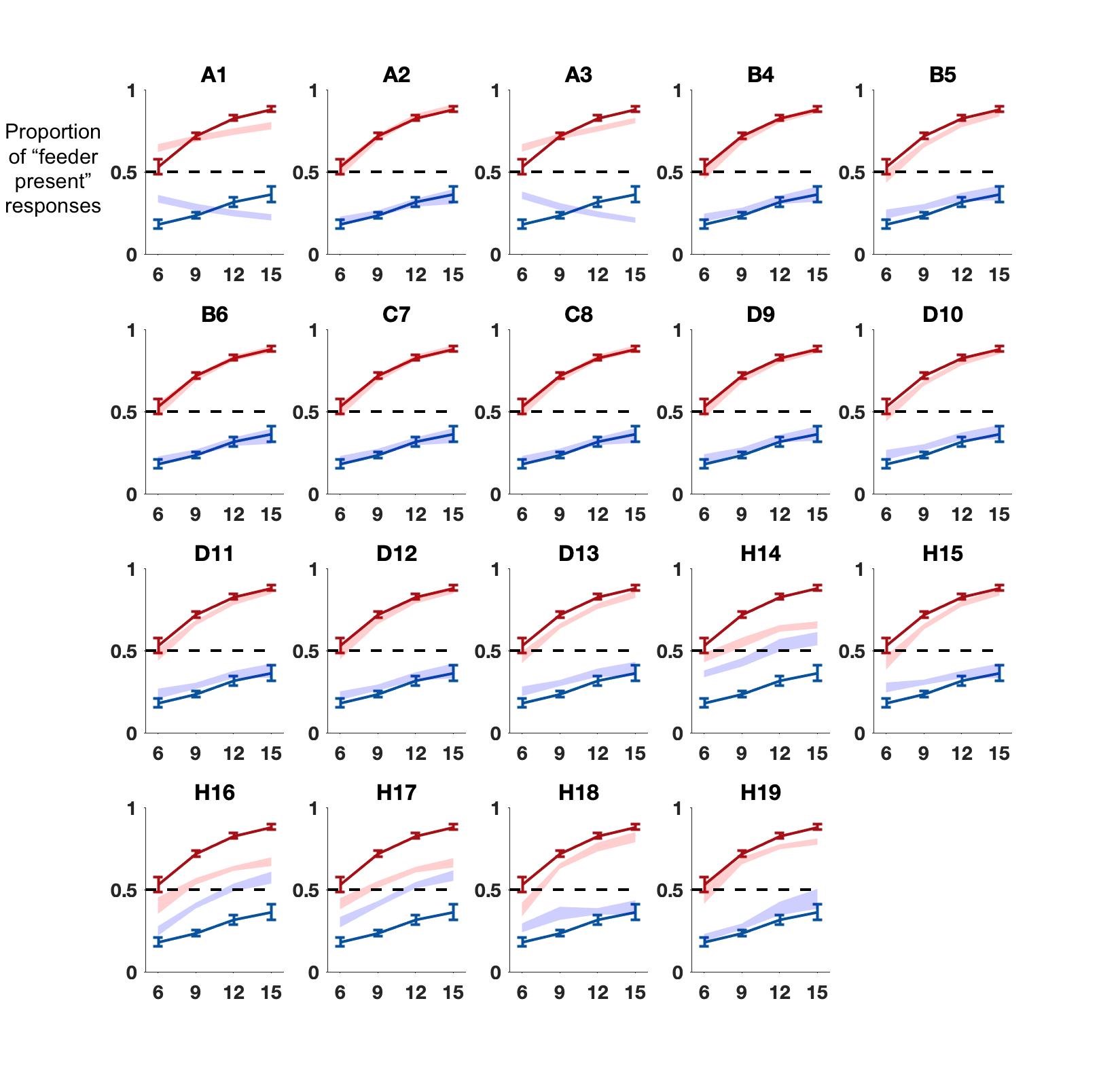

Supplement: S7 Fig — Model fits of proportion of “feeder present” responses as a function of number of pigeons (N), denoted by shaded area; subject data denoted by solid lines. (TIFF) [file pcbi.1009159.s007.tiff]

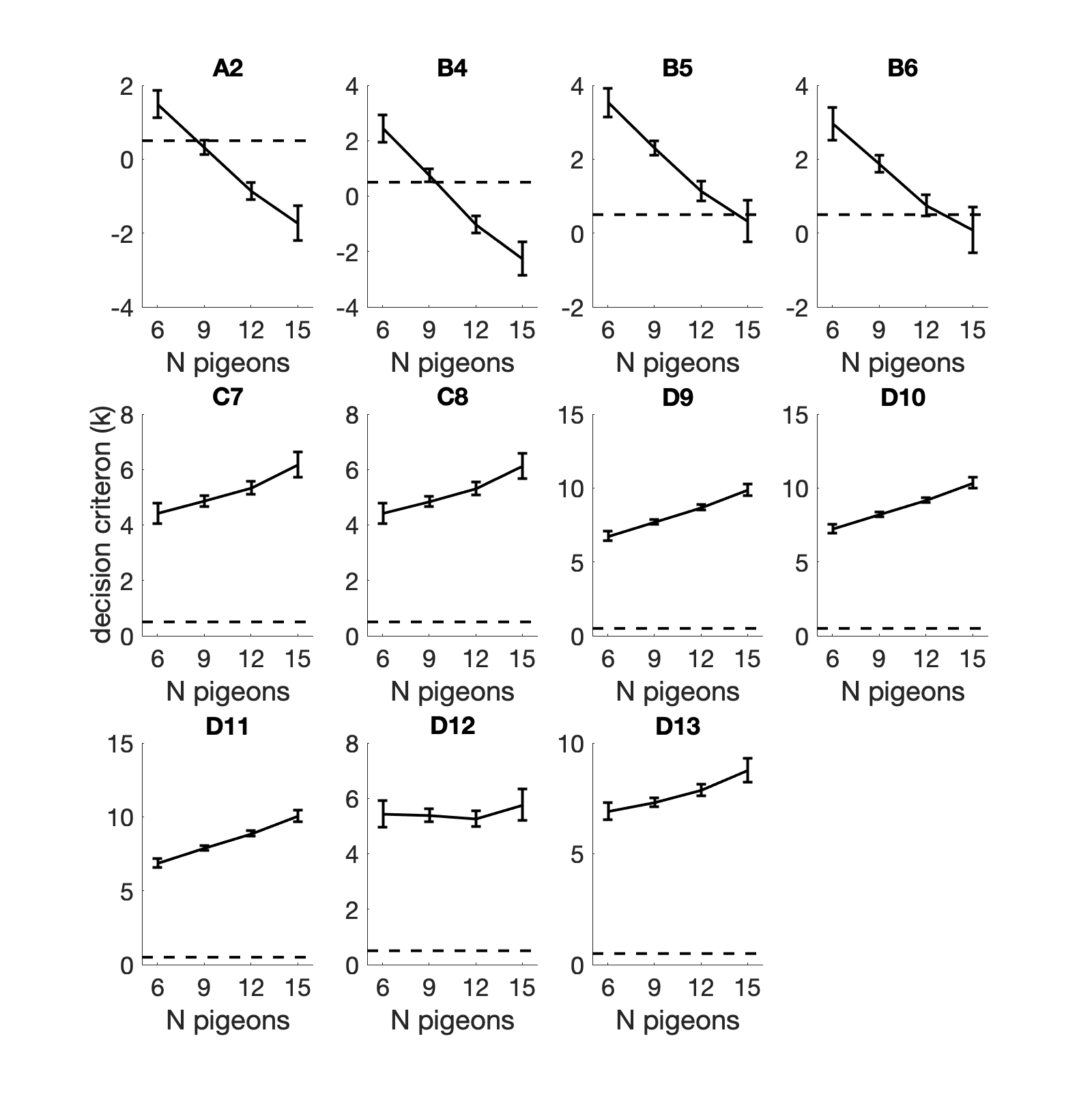

Supplement: S8 Fig — Value of the fitted decision criterion parameter kN for each N, shown for each of the basic models. (TIFF) [file pcbi.1009159.s008.tiff]

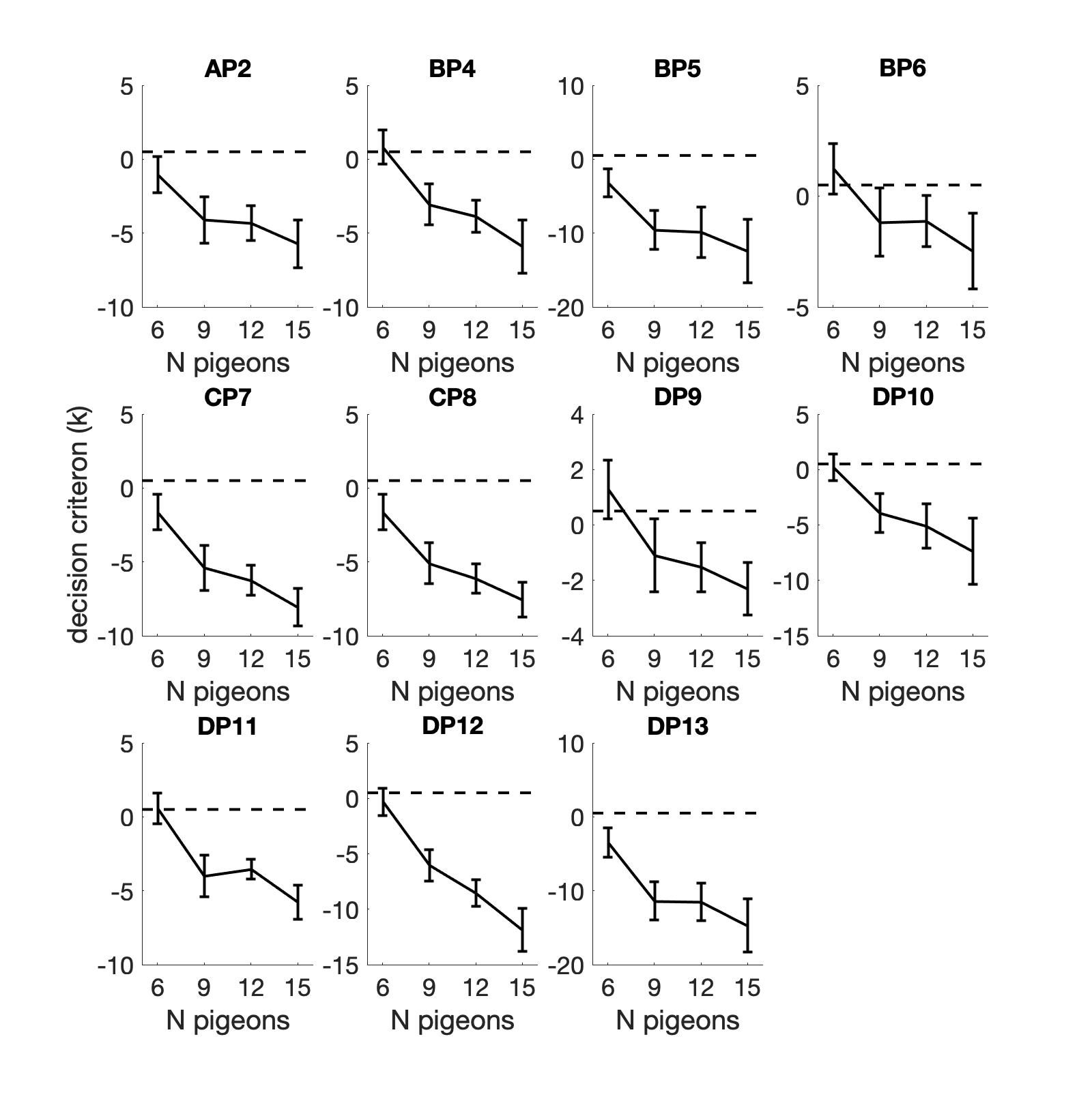

Supplement: S9 Fig — Value of the fitted decision criterion parameter kN for each N, shown for each of the model variants with a flexible paff. (TIFF) [file pcbi.1009159.s009.tiff]

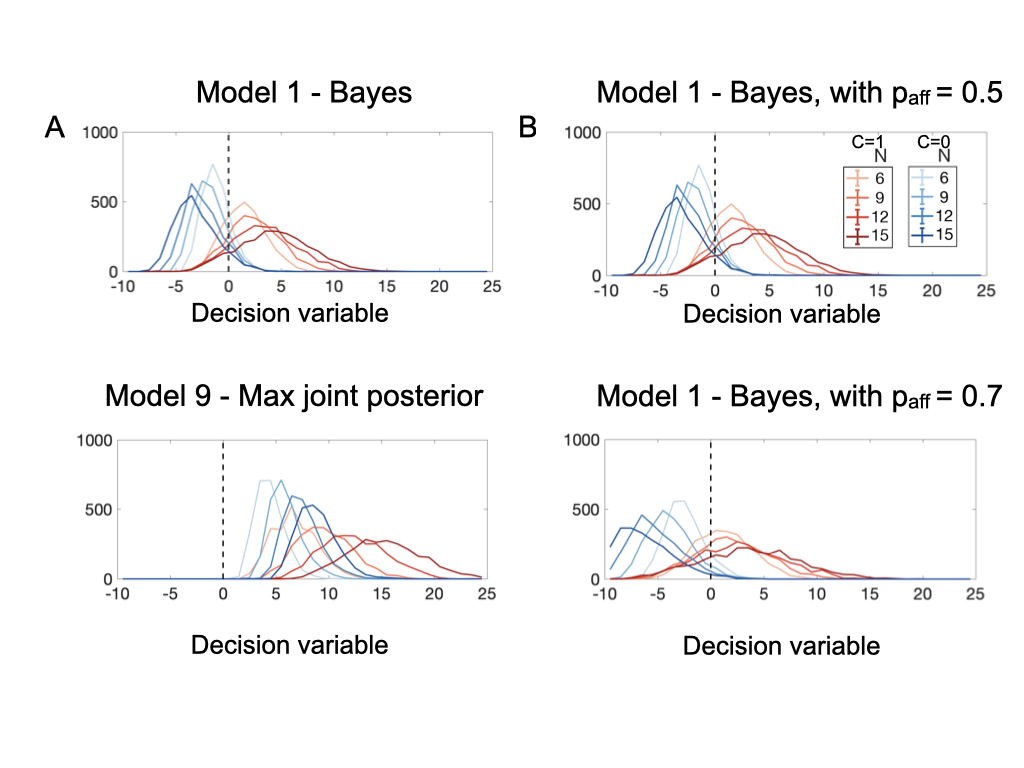

Supplement: S10 Fig — Histograms of decision variable distributions for the Bayesian model (paff = 0.5) in the top row, with two examples of shifted decision variable distributions. Panel A: maximum joint posterior (model 9, right-shifted). Panel B: Bayesian model with a false probability of affiliation of 0.7 (left-shifted). (TIFF) [file pcbi.1009159.s010.tiff]

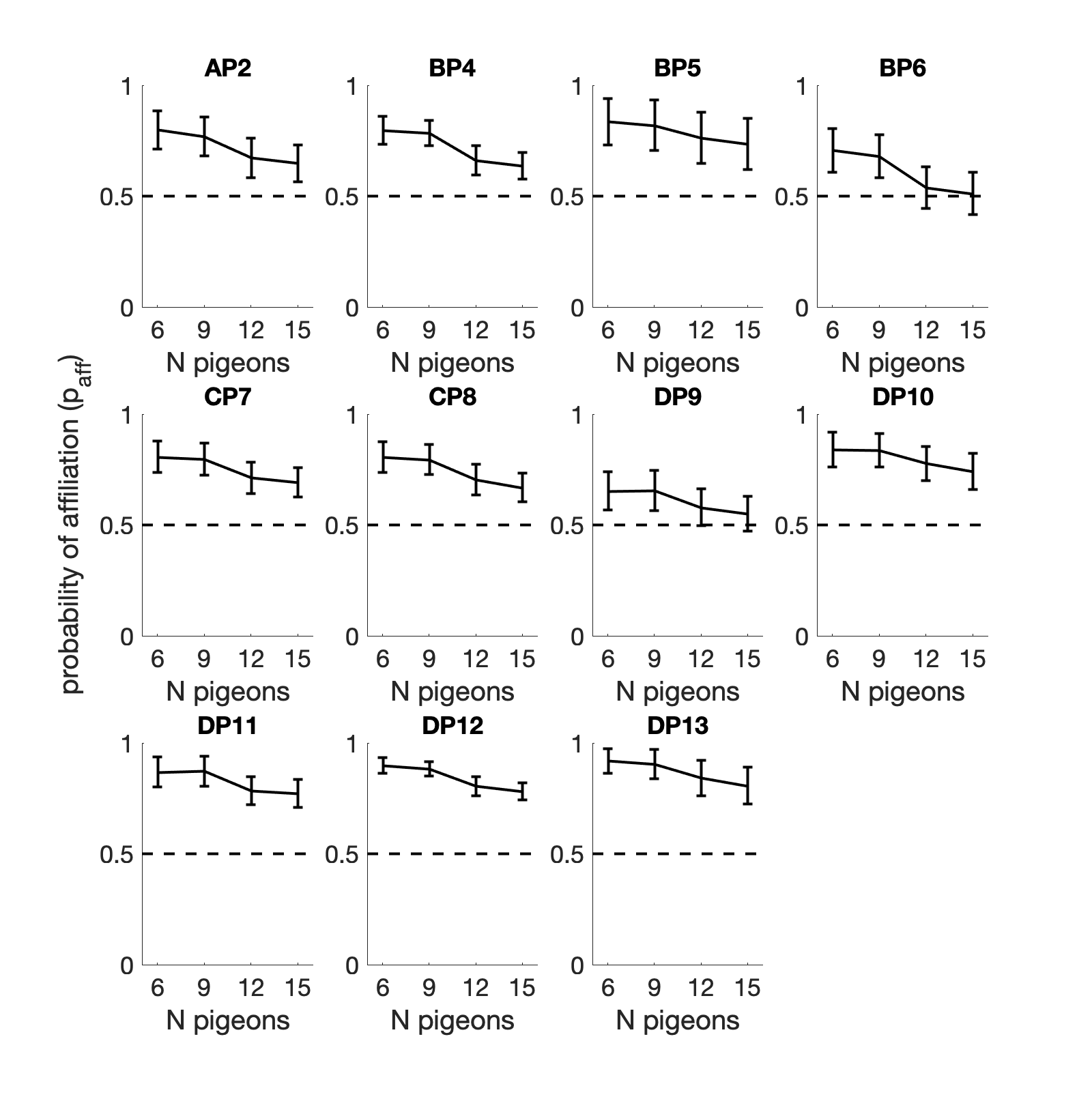

Supplement: S11 Fig — Fitted paff for each N shown for each model variant. (TIFF) [file pcbi.1009159.s011.tiff]

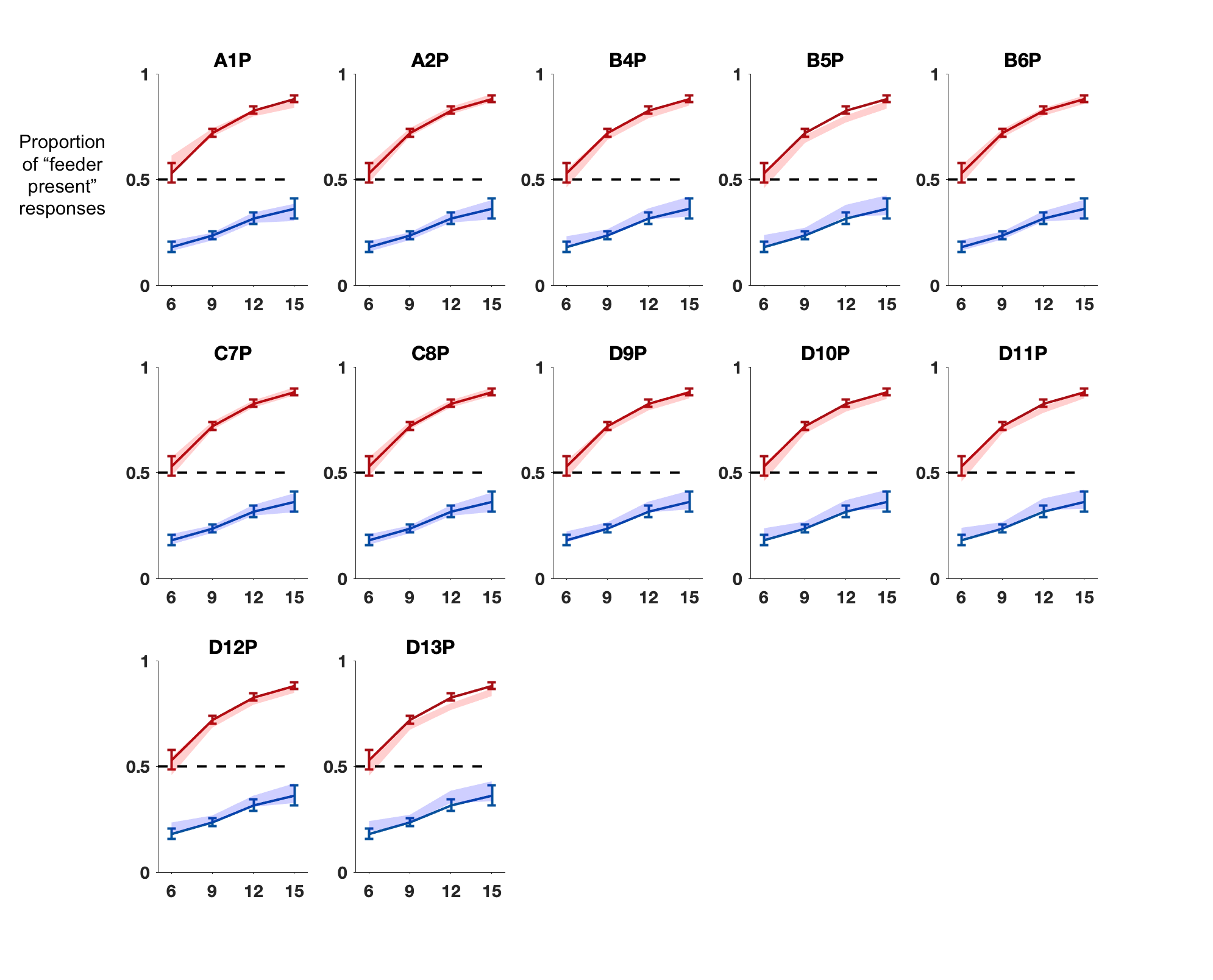

Supplement: S12 Fig — Model fits of proportion of “feeder present” responses as a function of number of pigeons (N), denoted by shaded area; subject data denoted by solid lines. (TIFF) [file pcbi.1009159.s012.tiff]
