# Supplementary material for: Point-estimating observer models for latent cause detection
Source: PLoS Comput Biol. 2021 Oct 29;17(10):e1009159. doi: 10.1371/journal.pcbi.1009159 (PMC8580258; doi:10.1371/journal.pcbi.1009159)
Supplement: Attachment — Submitted filename: Pigeon reviewer response.pdf [file pcbi.1009159.s013.pdf]

We thank both reviewers for their comments and address each point below.

Reviewer #1: The authors introduce and describe a novel experimental paradigm for inferring the presence of a ‘food feeder in a park’ where some nuisance variables (location of the feeder and pigeon association) make exact inference intractable. Then, the authors compare both Bayesian and point-estimating models using model comparison. It looks that some models involving marginalization over the latent variables cannot be rejected based on the choice data alone, so I think that some of the statements (such as the title) could be a little bit interpretative and not directly supported by the data. Nevertheless, the work is very strong in comparing models that marginalize or make assumptions to simplify computations, and thus it is of high value.

Thank you for these positive comments. We take “point-estimating” observer models to include even those that involve marginalization over latent variables so long as there is also a point-estimate involved for at least one of the latent variables. We emphasize point-estimating observers in the title to highlight the surprising fact that many of these models, which represent a latent variable as a point, rather than a full probability distribution, provide indistinguishably good fits to behavioural data.

It seems that perfect Bayesian estimation is contrasted with point estimation. Another way to simplify marginalization is based on the use of priors that highly constraints the size of the sum to the carried. If my reading is correct, no model in the paper uses some form of priors, and it might be unclear why they are not used within the general context of testing multiple models simultaneously.

In all models, we assume p(C=0) = P(C=1) = 0.5; in other words, all of the models are assumed to have equal or uniform prior belief that the feeder is either present or absent. Changing the priors to bias one response over another would be equivalent to changing the criterion *k*, which is already fitted as a free parameter in all models-- see Fig 11. Moreover, changing the prior over category does not constrain the computational complexity of the marginalization problem, which, in this case, arises from the calculation of the likelihood ratio.

One might think that using a very narrow class-conditioned stimulus distribution (e.g. p(mu|C=1)) could help constrain the complexity of the marginalization problem. Indeed, we do test for a flexible variant of the Bayesian model in model A3, in which the variance of the normal distribution from which pigeons are drawn (i.e. the class-conditioned stimulus distribution) is assumed not to have been correctly learned by subjects (and is thus a fitted parameter). This model does quite well and is a significant improvement on the true Bayesian model A1 (Fig 9) but does not perform as well as other models that assume the correct class-conditioned stimulus distribution with different computational steps.

Traditionally, the London bombing problem has been treated in a different way, in my understanding: one looks at the distribution of counts per bin, and tests whether the distribution is Poisson or not. Would something like this (which is a classical test) be something that participants might be using?

This traditional test, employed by Clarke's original article on the London Bombing problem (Clarke, R. D. (1946). An application of the Poisson distribution. *Journal of the Institute of Actuaries*, *72*(3), 481-481), drops the spatial arrangement of points. The way Clarke describes the data before doing any tests at all requires discarding their spatial location. In reality, there is a coupling between count and spatial coordinates. For instance, Clarke's test is insensitive to the distance between two points so long as they lie in different bins. This test is one of many heuristic tests that people could be employing to detect deviations from non-uniformity. We believe the heuristic models we have tested (e.g. mean density and local density, with a fitted decision variable) are similar in nature to these kinds of frequentist tests, and based on spatially sensitive statistics. In these heuristic cases, there is a frequentist statistical test that has to be done at the end (eg in Clarke's approach, a chi-squared test determines whether the expected vs. observed distribution is sufficiently different); we provide similar heuristic models where the test threshold is fitted.

The effects of N in Fig. 3 are very strong and relevant. Recent work by Schustek and Moreno-Bote (Nat Communications, 2019) show also a very strong effect of sample size N on responses and confidence reports, thus supporting the notion that subjects do not use too simple heuristics to solve hard inference problems. This paper could be commented in the discussion.

We agree that this is a relevant paper and have added the reference to the discussion section.

"In context of this complex latent cause detection task, the effect of the number of observations on false alarm rates was strong. Recent work tested the ability of subjects to infer a hidden low-level variable (the proportion of red vs. blue team supporters leaving a given airplane) based on both observations of differently sized samples, and an inferred higher-level contextual variable (the general red or blue-team bias for a set of planes). \cite{schustek2019human} show that inferred context is integrated with observations to inform confidence estimate, and notably that the sample size of previous observations guides context reliability. The sample size of observations may therefore be a factor that subjects take into consideration when performing hierarchical latent variable inference in the real world, affecting both choice and confidence judgements. In the case of our study, however, subjects suboptimally set a more liberal detection criterion as sample size increased, perhaps reflecting certain real-world prior beliefs that a greater number of observations provides evidence for the presence of a latent spatial cause, regardless of the distribution of the observations."

Related to the above, the sample size used goes up to N=16, well above the subitizing regime, but models do not seem to incorporate the possibility of numerosity estimation errors.

We fit a different decision criterion k for every N condition, which suggests that subjects do not necessarily need to have precise numerosity estimations. However, our flexible-k models do leave open the possibility that subjects are able to tell apart trials with different N (to be able to set an N-dependent decision criterion).

The agglomerative clustering algorithms looks a good model candidate due to its simplicity (although other models that require marginalization are as good as this one, as the authors

indicate). This model seems to be sequential in the way clusters are built, which means that studying eye movement could be a very relevant direction to be explored. Is not model comparison rather limited by the use of choice responses, with no RTs, gazes or confidence estimates?

These are all great suggestions which we will add to the discussion. However, we were not able to model RTs, gaze, and confidence estimates for the following reasons:

Reaction times: The agglomerative clustering algorithm is the only sequential or iterative model that makes a timecourse prediction, thus while we show that reaction time trends are well predicted by this model, we are simply not able to compare it to other models on the basis of RT predictions.

Eye-tracking: We accept that it is further a limitation of our study that we did not record eye-tracking behaviour, which might have helped adjudicate between different models by providing an indirect measure of the mu-estimate.

Confidence ratings: We show that, qualitatively, the confidence ratings do track the decision variables (log likelihood ratios) predicted by most of the top models (Fig 15, supplementary). However, our models already have many free parameters (especially those with the p_aff parameters), so in the interest of reducing the number of free parameters, we excluded confidence data from the model fits-- for instance, for 8 confidence levels, we would need an additional 7 decision criteria boundaries, potentially 7 x 4 boundaries to account for the 4 N conditions. We added a sentence in the methods section that explains why we chose not to model confidence responses.

“We did not design our models to explicitly account for confidence responses. Modelling confidence predictions for each of the 8 possible confidence levels would have required an additional 7 decision boundaries, possibly multiplied by the 4 N conditions, which would have introduced a large number of extra parameters without contributing additional understanding. Therefore, we chose to fit to choices only and subsequently make a zero-parameter prediction for confidence ratings.”

Reviewer #2: In this paper the authors provide a very thorough set of analyses and comparisons to test a range of models of the computations that might be going on within subjects in a cognitive task. The task is a great example of the sorts of computations all animals must do in every day life, while being simple enough that the potential solutions can be analyzed. The work of the authors is very rigorous, perhaps to the point that so many possibilities are being compared that the main point/thread can be lost amidst the large number of results. Therefore, since I think the work is valuable, my suggestions/critiques revolve around increasing readability, in particular, by highlighting and explaining the important findings more.

Specific comments:

Given the number of results and figures I think it is important they are cited clearly in the text in order, alongside a sentence giving the main finding of the figure. For example I do not see

Figures 5 or 6 cited in the text and others appear to be cited out of the numerical order in which they appear.

Figure 5 is already cited in the main text; figure 6 was originally referenced in the main text where the Agglomerative clustering algorithm is introduced and has been moved down to reflect the order in which it is cited.

Some of the figures, specifically Figs 8 and 9 along with the corresponding styles in the supplement have so many lines that the text is hard to read and to scan across to the corresponding curve. I suggest a bit more spacing between rows and clearer – probably larger – fonts are needed.

We took out redundant text to make the figure narrower and increased the size so it is a bit clearer. Thanks for the suggestion.

p.2 the sentence explaining nuisance variables would benefit from being broken down and expanded. E.g. What is the “variable of interest” (an example would be nice – I’m still not sure what it means in the actual experiment, presumably the binary variable “C”). It would also not hurt to add a sentence explaining how a “generative model” is not just any model, since it is a bit of a buzzword/phrase these days.

We have added further explanations of what is meant by “variable of interest” and “nuisance variable” in the context of the London bombing example to help clarify these concepts to the reader. (line 81 overleaf)

p.7 “following a bimodal distribution” – please explain why “feeder present trials” would have a bimodal distribution of the numbers of affiliated pigeons, since the process appears to be Binomial which has a unimodal distribution.

Thank you for pointing out this error- we meant to write “binomial” instead of “bimodal.”

p.8 “one strong contender for winning model” – I guess you mean the model that best fits the data rather than the one that results in optimal performance? (also either “a” or “the” is missing).

Yes! Changed to clarify that they each present at least one strong contender for “the model that best fits subject data.”

Figure 6: even for feeder absent trials the log-likelihood is mostly above zero indicating feeder present in my understanding. I think this is why the “decision threshold” needs to be optimized to match the data, but this seems like a big problem with this and many of the other models that should be stated clearly (if you expected this then say why) and discussed in the discussion. In particular in Supp Fig 18 it seems that for whole families of models the decision criterion is either always positive or always negative – to help the reader these qualitative changes across models should be explained. i.e. there must be a reason why some models are biased toward positive and others to negative likelihood ratios. The explanation on p.16 (e.g. different motor costs) is unsatisfactory as that would be revealed in a behavioral bias rather than a model bias and in any case does not say why different families of models would err in different directions (or why does the Bayesian model need a specific N-dependence, from the point of view of what is going on within the model, not just the observed output). That is to say, irrespective of behavior, what is going on in the models

to require such biases? Or are different “d” values only needed in the model to explain behavioral biases and all models produce greater accuracy with a d of zero? A lot more explanation is needed concerning this issue.

There are multiple factors that contribute to the decision criterion. One factor is, in some models, we are singling out particular mu’s or z’s that are particularly good-- thereby biasing the log posterior ratio estimates to higher values. Overall positive decision criteria might be expected if the mu’s and z’s that are being committed to are quite good-- for example, Model 9 evaluates the LLR at only the mu and z that maximizes the joint posterior, which means that the estimated LLR for a given trial will shift upwards. See figure panel A.

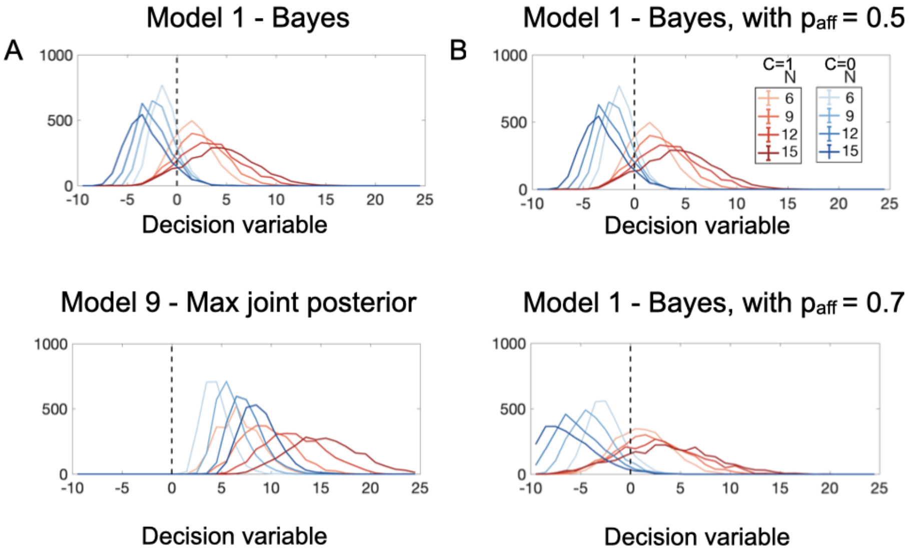

Similarly, for models with p_aff, if p_aff is higher than 0.5, then it is optimal to shift the decision variable down, since the distribution of decision variables for trials of both categories will shift down. This is because, if subjects mistakenly believe that the probability of affiliation for the C=1 (feeder present) hypothesis is higher than it is, then the estimated log posterior ratio on a given trial will be lower (towards C=0, feeder absent). Conversely, if the probabiltiy of affiliation is believed to be lower than 0.5, then we bias the decision variable distributions upwards, towards a C=1 (feeder present) response. Compare the histograms in figure panel B, showing the leftward shift of the decision variable distributions when the observer believes the probabilty of affiliation is 0.5 (top) vs. 0.7 (bottom).

Lastly, there are other models that are mixed because the committed mu’s and z’s are not particularly good.

The decision variable’s zero point is only meaningful for the Bayesian model, because a perfect Bayesian observer should set their decision criterion to zero. However, zero does not mean much on non-Bayesian decision variable estimates-- at the point at which observers are already estimating the log posterior ratio, it’s conceivable that they would set a different decision threshold more appropriate for that decision variable scale. E.g., for point-estimate models where we select a highly probable mu, we are biasing the log posterior ratio distribution by no longer considering all the possible mus. Relative to the Bayesian decision variable on a given trial, the point-estimate observer decision variable will be

For some models, it is obvious why the decision variable does not go below 0. For example, for a purely heuristic model that calculates the mean density of points, mean density is a

heuristic decision variable that can only take on a positive value. The absolute value of the decision variables are therefore not particularly interesting or meaningful, since we fit the decision threshold per subject, except for the case of the true Bayesian model, in which the decision threshold should normatively be set at 0, since its decision variable is the log posterior ratio.

Added to the main text:

Supplementary Fig. \ref{fig:ks} shows fitted decision criteria for each model across subjects. There are a number of factors that contribute to the fitted decision criterion across models-- for instance, the goodness of the committed variables, and the subject's fitted p_{aff} beliefs. Some models commit to a particular μ or z that is particularly good, thereby biasing the log posterior ratio estimates to higher values. Overall positive decision criteria might therefore be expected if the committed variables are quite good-- for example, Model 9 evaluates the LLR at only the μ and z that maximizes the joint posterior, meaning the estimated LLR for a given trial will shift upwards (see supplementary Fig \ref{fig:didactic}, panel A). Similarly, models with $p_{aff} > 0.5$ may cause the decision variable distributions (and therefore the fitted decision criteria) to shift downwards. If the probability of affiliation for the feeder present ($C=1$) hypothesis is higher than 0.5, then it should take a greater amount of dot ``clustering'' to support the feeder present hypothesis, and the observer's decision variable on a given trial will shift lower, in the direction of the feeder absent ($C=0$) hypothesis (see supplementary Fig \ref{fig:didactic}, panel B). Note that the decision variable's zero-point is only meaningful for the perfect Bayesian model (since a Bayesian observer should set their decision criterion to zero), whereas observers likely set a different decision threshold when the log posterior ratio is estimated (as in all other models), in compensation for the fact that these estimates produce shifts in both $C=0$ and $C=1$ distributions.

Discussion: I would like to see a bit more focus on what is new/surprising, why some calculations are feasible in the brain, not others, what calculations (e.g. agglomerative clustering) have any evidence for them in other literature and so on. Are there ways of producing stimuli more artificially that would hinder one of the strong contender models more than another, and so on. As of now it does not seem like there is a strong takeaway. My understanding is that the full Bayesian method would be intractable anyway so I am not sure it was ever a serious contended, but here it is good to see some evidence disfavoring it. But again, some more discussion about the “false priors” results is needed. Do these really rescue the Bayesian model? Is there any reason to think that the subject would know the exact priors so not produce false priors? If not, perhaps the “false priors” are optimal given the data in some sense, so would that not make them a “more Bayesian” version than the standard?

We think it is already a strong takeaway, and appropriately emphasized in the discussion, that point-estimating observers constitute an overlooked class of models, and that approximate Bayesian inference while simplifying certain computationally taxing steps. While the full Bayesian model may be intractable in some perceptual tasks, it has been shown to be a serious contender in many others, so our work contributes by helping to define which kinds of tasks on which the Bayesian model may perform poorly, to help narrow down the

source of possible limitations in human decision-making. Namely, we propose that Bayesian models may perform poorly on tasks where the perceptual decision-making problem requires marginalization over an explosive number of nuisance parameters, and we propose that point-estimating observers constitute a class of models that may perform better on such tasks.

Regarding “false priors”: Allowing for the decision criterion to be fitted to each subject leaves open not only the possibility of “false priors” but also a number of other reasons why a decision criterion might deviate from 0. The subject might, for instance, have a different cost associated with getting an answer right in one direction vs. another (for instance, it may be somehow more subjectively rewarding to produce a false positive compared to a false negative result, even if both are simply considered wrong). Fitting a different decision criterion by N also helped demonstrate that subjects may in fact be biased towards identifying a causal pattern the more observations exist, which is interesting in and of itself. It will have to remain an open question for another study why this bias (towards identifying patterns as observations increase) may actually allow for optimal perception in the real world, or may have developed in order to optimize some global perceptual decision-making problem (e.g. perhaps in the real world, there are asymmetric stakes in pattern identification that causes people to respond more liberally when there are a greater number of observations).

As you can see, with the wealth, even overabundance, of tests and results, a lot of questions are raised and the paper would be more satisfactory if a sizable subset of potential questions are discussed and addressed in the text.

Finally, looking at the GitHub repository with the code, the ReadMe is insufficient to help anyone run the code (there are so many files I suspect it would be practically impossible for anyone to run it, so without instructions it is in practice unavailable).

We have added instructions for running the files to be able to fit models and create the visualizations included in this manuscript.

Minor issues:

p. 5 “Family B” is used when I think you are still describing Family C (right after the eq.).

Fixed

top of p.12 a weird symbol instead of “<” for the p-value

Fixed

Eq.23: I think N_0 and N_1 should be defined though one can guess they are numbers non/affiliated with the feeder in a given trial.

Fixed

Before eq.24 a missing close parenthesis

Fixed

p.20 “see … box” it would be nice to tell the reader where to find the box as it is not nearby.

Fixed

Have the authors made all data and (if applicable) computational code underlying the findings in their manuscript fully available?

The PLOS Data policy requires authors to make all data and code underlying the findings described in their manuscript fully available without restriction, with rare exception (please refer to the Data Availability Statement in the manuscript PDF file). The data and code should be provided as part of the manuscript or its supporting information, or deposited to a public repository. For example, in addition to summary statistics, the data points behind means, medians and variance measures should be available. If there are restrictions on publicly sharing data or code —e.g. participant privacy or use of data from a third party—those must be specified.

Reviewer #1: Yes

Reviewer #2: **No:** See above -- present but impractical to use without instructions.

More detailed instructions have been added to the readme file.

PLOS authors have the option to publish the peer review history of their article (what does this mean?). If published, this will include your full peer review and any attached files.

If you choose “no”, your identity will remain anonymous but your review may still be made public.

Do you want your identity to be public for this peer review? For information about this choice, including consent withdrawal, please see our Privacy Policy.

Reviewer #1: No

Reviewer #2: No

Thank you both very much for your thoughtful feedback, and we hope these changes have sufficiently addressed your concerns.

Jennifer Lee

Wei Ji Ma
